# Ethnic Differences in Tissue Creatine Kinase Activity: An Observational Study

**DOI:** 10.1371/journal.pone.0032471

**Published:** 2012-03-16

**Authors:** Lizzy M. Brewster, Carmen M. D. Coronel, Willem Sluiter, Joseph F. Clark, Gert A. van Montfrans

**Affiliations:** 1 Departments of Internal and Vascular Medicine, Academic Medical Center, University of Amsterdam, Amsterdam, The Netherlands; 2 Department of Pathology, St. Elisabeth Hospitaal, Willemstad, Curaçao; 3 Center for Lysosomal and Metabolic Diseases, Erasmus Medical Center, University of Rotterdam, Rotterdam, The Netherlands; 4 Vontz Center for Molecular Studies, University of Cincinnati, Cincinnati, Ohio, United States of America; Université Joseph Fourier, France

## Abstract

**Background:**

Serum creatine kinase (CK) levels are reported to be around 70% higher in healthy black people, as compared to white people (median value 88 IU/L in white vs 149 IU/L in black people). As serum CK in healthy people is thought to occur from a proportional leak from normal tissues, we hypothesized that the black population subgroup has a generalized higher CK activity in tissues.

**Methodology/Principal Findings:**

We compared CK activity spectrophotometrically in tissues with high and fluctuating energy demands including cerebrum, cerebellum, heart, renal artery, and skeletal muscle, obtained post-mortem in black and white men. Based on serum values, we conservatively estimated to find a 50% greater CK activity in black people compared with white people, and calculated a need for 10 subjects of one gender in each group to detect this difference. We used mixed linear regression models to assess the possible influence of ethnicity on CK activity in different tissues, with ethnicity as a fixed categorical subject factor, and CK of different tissues clustered within one person as the repeated effect response variable. We collected post-mortem tissue samples from 17 white and 10 black males, mean age 62 y (SE 4). Mean tissue CK activity was 76% higher in tissues from black people (estimated marginal means 107.2 [95% CI, 76.7 to 137.7] mU/mg protein in white, versus 188.6 [148.8 to 228.4] in black people, *p* = 0.002).

**Conclusion:**

We found evidence that black people have higher CK activity in all tissues with high and fluctuating energy demands studied. This finding may help explain the higher serum CK levels found in this population subgroup. Furthermore, our data imply that there are differences in CK-dependent ATP buffer capacity in tissue between the black and the white population subgroup, which may become apparent with high energy demands.

## Introduction

Creatine kinase (CK) activity in serum is widely used to diagnose tissue damage including myocardial infarction and skeletal muscle myopathy, but it is unknown why serum CK activity is higher in apparently healthy black people of sub-Saharan African descent [1]–[4]. In our population study we found a median activity in serum CK of 88 IU/L in white people and 149 IU/L in black people using random sampling [1]. There is no evidence of muscle damage in black people as cause for the high serum CK activity, and the BB, MB, and MM isoenzymes in serum are proportionally higher, but have a normal distribution [2], [4]. Serum CK in healthy subjects is thought to be derived from normal tissue “leaking” CK to lymphatic vessels and into the blood stream, proportionate to the intracellular CK concentration [3]. Therefore, it was proposed that the black population subgroup has a generalized high CK activity in tissues, but hitherto there were no data to substantiate this [3]. In this paper, we report ethnic differences in CK activity in tissues with high and fluctuating energy demands.

## Methods

### Collection of tissues

Tissues were collected in accordance with ethical approval of the accredited Medical Ethics Committee of the Academic Medical Center, Amsterdam, the Netherlands, whereby post-mortem small sample tissue collection was permitted with written consent from the donor, or a legal representative (next-of-kin or other authorized representative). We only included tissues of subjects presented within 30 h after death. Exclusion criteria included known HIV, hepatitis B, or C infection; Creutzfeldt-Jacob disease; chronic heart failure; myocardial infarction; brain infarction; or any widespread disease of the sampled organs.

Ethnicity of the deceased was determined based on chart review of self-identification, country of birth, or information from next-of-kin indicating which ethnicity the decedent considered himself to be, if available, and phenotypic traits such as skin colour (including the extremes of Fitzpatrick natural skin tone type I/II for white, vs Type V/VI for black people [5]), and other physical features. Classification in black of sub-Saharan Africa or of sub-Saharan African descent, or white European was final when consensus was reached by at least two health workers (pathologists, a member of the study group, or pathology assistants).

Around 1 cubic centimeter of macroscopically healthy cerebral cortex (gyrus precentralis), cerebellar cortex, left cardiac ventricle, renal artery, and iliopsoas muscle was sampled. Tissue samples were snap frozen in liquid nitrogen and stored at −80°C until the estimation of enzyme activity.

### Creatine kinase determination

#### Tissue homogenization

Snap-frozen tissue samples were weighed, transferred into ice-cold SHE buffer (250 mM sucrose, 10 mM HEPES, 1 mM EDTA, pH 7.4) at 5% w/v, minced and homogenized on ice in a pre-cooled Potter-Elvehjem glass tube by 12 maceration strokes up and down with a tight fitting Teflon pestle rotating at 800 rpm. The homogenate was snap-frozen in liquid nitrogen and stored in small aliquots at −80°C until the assay was performed.

#### Biochemical assays

The investigator was blinded for the ethnicity of the subjects. CK activity was determined according to Scholte et al. [6], at 25°C with 1.5 µM rotenone and 7.5 µg/mL oligomycin added to the assay mixture, and the concentration of EDTA and pyruvate kinase adjusted to respectively 2 mM, and 2 U/mL. The baseline reaction rate was spectrophotometrically assessed (Cary 1E Spectrophotometer; Varian Inc., Middelburg, The Netherlands), for 3 min at 340 nm, before the reaction was started by adding 25 mM creatine, and followed for 5–10 min. The reaction rate was corrected for the baseline rate, and CK activity was calculated using a molar extinction coefficient of NADH of 6.3 cm^2^/µmole, and expressed in U/ml sample. The protein content of the homogenates was determined with Bovine Serum Albumin as a standard protein by the Bio-Rad DC protein assay (Bio-Rad Laboratories, Inc., Veenendaal, The Netherlands), which is a modified Lowry assay [7].

### Statistical analysis

#### Primary outcome

The primary outcome was a difference in mean tissue creatine kinase activity between black and white people.

#### Sample size calculation

Based on previous reports, which indicated an up to two-fold higher CK activity in serum of black people [1]–[4], we conservatively estimated to find a 50% greater mean CK activity in tissues of black people compared to white people, and calculated a need for 10 subjects of one gender in each group to detect this difference with a one-tailed alpha of 0.05 and a 1–beta of 0.80.

#### Data analysis

Correlations with tissue CK activity were calculated using Pearson's correlations coefficients, for storage time, ethnicity, and for age nested within ethnicity. We used mixed linear regression models to assess the possible influence of ethnicity on CK activity in different tissues, with ethnicity as a fixed categorical subject factor, and CK of different tissues clustered within one person as the repeated effect response variable.

Taking ethnic differences in the mean tissue CK of different tissues clustered within one person as the response variable in mixed linear regression modelling (rather than testing pairs of tissue types separately with a Student *t* test), is just, as we assumed the outcomes for tissue types within one person to be highly correlated. In addition, we hereby greatly reduced the required sample size, which was of vital importance to be able to perform this type of study, as in our country, subject infrequently opt to donate post mortem tissue samples for scientific studies. Age was nested within ethnicity in the model. For the final model, we examined 3 covariance structures (diagonal, autoregressive, and compound symmetry), using Akaike's Information Criterion and Schwarz's Bayesian Information Criterion.

A one-tailed *p* value of 0.05 or less was considered statistically significant. Data were analyzed with SPSS statistical software package for Windows, version 16.0 (SPSS Inc., Chicago, IL, USA).

## Results

We included 27 subjects, 10 black men of African-Caribbean descent and 17 white men, with a mean age of 62 y (SE 4). Tissues were collected within a mean of 26 h (SE 3.0) after death (black people 26 h (SE 2.1), white people 25 h (SE 4.8), *p* value for the difference = 0.9).

**Figure 1:**
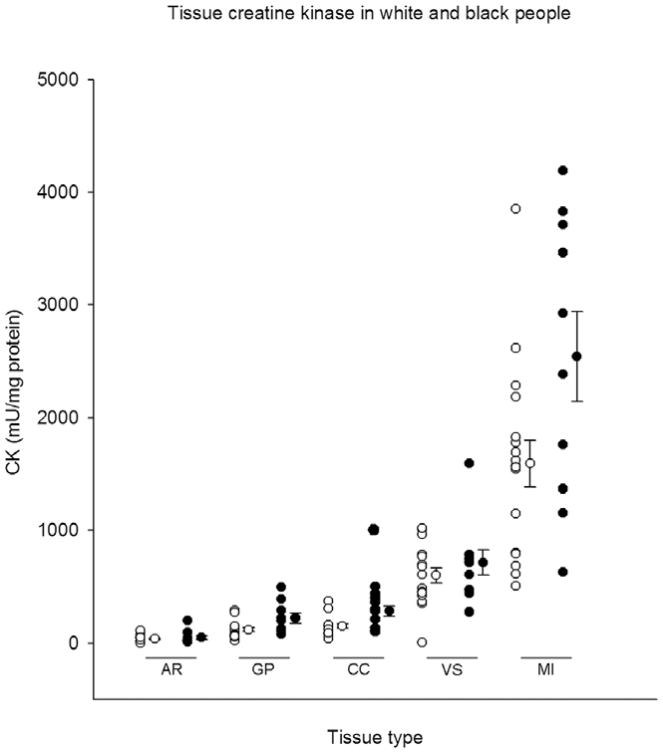
Tissue creatine kinase in black and white people. CK, creatine kinase. AR, arteria renalis; GP, gyrus precentralis; CC, cortex cerebelli; VS, ventriculus sinister cordis; MI, m. iliopsoas. Open and closed circles depict CK activity (mU/mg protein) in white and black subjects respectively, with the bars representing the mean CK activity per ethnic group and tissue type with the standard error.

There was no significant correlation between storage time and mean tissue CK activity, nor between age and CK activity. We did find a significant correlation between CK and ethnicity (correlation coefficient 0.43; *p* = 0.025). In the regression analysis, Type III tests of the fixed effect of ethnicity on CK activity in different tissues (**Figure 1**) was significant (*p* = 0.002), with a diagonal covariance structure as the most appropriate model. The post hoc pairwise comparison of the least square means for different levels of the fixed factor ethnicity, showed a 76% higher mean tissue CK activity in black people, respectively 107.2 [95% CI, 76.7 to 137.7] mU/mg protein in white people, versus 188.6 [148.8 to 228.4] in black people.

## Discussion

This study provides evidence that CK activity in different types of tissues with high and fluctuating energy demands is higher in black people than in white people. Serum CK activity is thought to be resultant of tissue CK concentration, the release of CK from tissues, lymphatic flow, and CK clearance by the liver, as reviewed by Brewster et al. [3]. Normal tissue loses a small fraction of cytosolic CK into the interstitial space, as was shown in ^31^P nuclear magnetic resonance spectroscopy studies [8]. In physiologic and pathological states, release from tissue is proportional to tissue CK activity [3], [8]. Interstitial CK is subsequently transported through lymphatic vessels into the blood stream [3]. Consequently, the differences in tissue CK activity might be reflected in serum as well.

The higher CK activity we previously found in serum of black people in our population study (70%) [1] is proportionate to the higher activity in tissue (76%). Thus the unexplained high serum CK in healthy black people [1]–[4], with a normal isoenzyme distribution [2], [4] might be associated with a generalized high CK activity in tissues of this population subgroup.

CK is the central regulatory enzyme of energy metabolism [9]–[12]. The enzyme catalyzes the reversible transfer of the phosphoryl group (P) between creatine and ADP:

**MgADP+PCreatine+H^+^ ↔ MgATP+Creatine**

At subcellular locations with high energy demands, CK rapidly regenerates ATP, near Na^+^/K^+^–ATPase and Ca^2+^–ATPase at membranes, as well as near myosin light chain kinase and myosin ATPase at the contractile proteins. CK thus fuels highly energy-demanding processes such as cardiovascular contractility, sodium pumping, and trophic responses, at a faster rate than glycolysis and oxidative phosphorylation together [9]–[12].

High tissue activity of the CK system may alter the behaviour of the cellular energy metabolism system, leading to enhanced energy-dependent responses [3], [10], as well as to the emergence of new characteristics [13], such as a shift towards contractile responses in microvascular smooth muscle instead of the development of the latch state [3], [10]. In addition, creatine synthesis is closely linked to nitric oxide bioavailability through the common precursor, L-arginine [3]. As creatine synthesis demands the major portion of the available L-arginine, high tissue CK may drive the system towards a reduced bioavailability of nitric oxide [3].

The strength of this study is that we assess ethnic variation in CK in all relevant organs with high and fluctuating energy demands, including brain, heart, and renal arteries. These data may help explain the clinically important finding of a high serum CK with a normal distribution of isoenzymes in healthy black people [1]–[4].

We assessed CK in different tissues based on ethnicity, although race and ethnicity are much less objective and therefore more difficult to conceptualize and measure than parameters such as sex, age, or blood pressure [14]. However, race or ethnicity correlates well with health outcomes [3], [15], and with genetic clusters [16]. Because of these persistent associations of measures of race or ethnicity with health, it is important to continue studying these population subgroups, but to improve the ways in which these concepts are captured [14]. In this study, we stated state explicitly how ethnic classifications were defined and assessed, so that consistency in future studies can be maintained [14].

Finally, inherent to the types of tissue needed including cerebral tissue, our sample size is relatively small, but the differences are apparent at this sample size, in accord with our a priory power calculation. However, this work should be regarded as preliminary and requires confirmation in study of tissues of white and black populations in other centres.

In conclusion, we found evidence that black people have higher CK activity than white people in tissues with high and fluctuating energy demands. This may contribute to the higher serum CK levels with a normal isoenzyme distribution, found in the black population [1]–[4]. Further experimental study is needed to assess how human variation in the activity of the most fundamental of biochemical reactions, energy generation, may affect the capacity of the tissues to function under highly demanding conditions.
